# Preformed CD40L Is Stored in Th1, Th2, Th17, and T Follicular Helper Cells as Well as CD4^+^8^−^ Thymocytes and Invariant NKT Cells but Not in Treg Cells

**DOI:** 10.1371/journal.pone.0031296

**Published:** 2012-02-21

**Authors:** Yoshinobu Koguchi, Abigail C. Buenafe, Timothy J. Thauland, Jennifer L. Gardell, Elizabeth R. Bivins-Smith, David B. Jacoby, Mark K. Slifka, David C. Parker

**Affiliations:** 1 Department of Molecular Microbiology and Immunology, Oregon Health and Science University, Portland, Oregon, United States of America; 2 Department of Neurology, Oregon Health and Science University, Portland, Oregon, United States of America; 3 Division of Pulmonary and Critical Care Medicine, Oregon Health and Science University, Portland, Oregon, United States of America; 4 Vaccine and Gene Therapy Institute, Oregon Health and Science University, Beaverton, Oregon, United States of America; University of Nebraska Medical Center, United States of America

## Abstract

CD40L is essential for the development of adaptive immune responses. It is generally thought that CD40L expression in CD4^+^ T cells is regulated transcriptionally and made from new mRNA following antigen recognition. However, imaging studies show that the majority of cognate interactions between effector CD4^+^ T cells and APCs in vivo are too short to allow de novo CD40L synthesis. We previously showed that Th1 effector and memory cells store preformed CD40L (pCD40L) in lysosomal compartments and mobilize it onto the plasma membrane immediately after antigenic stimulation, suggesting that primed CD4^+^ T cells may use pCD40L to activate APCs during brief encounters. Indeed, our recent study showed that pCD40L is sufficient to mediate selective activation of cognate B cells and trigger DC activation in vitro. In this study, we show that pCD40L is present in Th1 and follicular helper T cells developed during infection with lymphocytic choriomeningitis virus, Th2 cells in the airway of asthmatic mice, and Th17 cells from the CNS of animals with experimental autoimmune encephalitis (EAE). pCD40L is nearly absent in both natural and induced Treg cells, even in the presence of intense inflammation such as occurs in EAE. We also found pCD40L expression in CD4 single positive thymocytes and invariant NKT cells. Together, these results suggest that pCD40L may function in T cell development as well as an unexpectedly broad spectrum of innate and adaptive immune responses, while its expression in Treg cells is repressed to avoid compromising their suppressive activity.

## Introduction

T cell help for APCs is essential for adaptive immune responses [1], [2]. Effector CD4^+^ T cells deliver help to antigen-specific B cells in an MHC class II-restricted manner [3]. CD40L, a membrane-bound cytokine in the TNF superfamily, plays a crucial role in this process. CD40L is also required for generating optimal CD4^+^ T and CD8^+^ T cell responses through activation of dendritic cells (DCs) [4]. Thus, lack of CD40L expression causes defective humoral and cellular immunity [5]. In contrast, dysregulated CD40L expression causes autoimmunity, lymphoma, and premature termination of humoral immunity [6], [7], [8], [9]. A recent clinical trial of recombinant CD40L failed to restore B cell responses whereas it successfully elicited Th1 responses in patients who harbor mutations in the genes encoding CD40L [10]. A precise understanding of CD40L regulation, including its expression and manner of delivery, could assist in the development of effective vaccines, immunological interventions for inflammatory diseases, and successful treatment of CD40L deficient patients.

It is generally thought that CD40L is synthesized from new mRNA (de novo CD40L) and delivered while effector CD4^+^ T cells are engaged in intimate interactions with cognate APCs in the time frame of a few hours [11]. This notion has been challenged by studies utilizing two-photon microscopy. While the initial, stable interactions of naïve CD4^+^ T cells and DCs can last for several hours, the majority of interactions between effector CD4^+^ T cells and cognate APCs in vivo are surprisingly short, ranging from several minutes up to 30 minutes [12], [13], [14], [15]. Although these short interactions are antigen-specific and presumed to be productive, 30 minutes is not enough time for effector CD4^+^ T cells to make de novo CD40L.

We propose that effector CD4^+^ T cells activate cognate APCs during brief interactions using preformed CD40L (pCD40L). We and others have demonstrated that human and mouse effector and resting memory CD4^+^ T cells retain pCD40L intracellularly, and that pCD40L can come to the cell surface within a few minutes of antigenic stimulation [16], [17]. Th1 cells store pCD40L in lysosome-related organelles known as secretory lysosomes [17], a category of secretory vesicles which includes the lytic granules containing perforin and granzyme B in cytotoxic T-lymphocytes (CTLs) and natural killer (NK) cells [18]. The existence of cytotoxic Th1 cells in humans and mice which resemble CD8^+^ CTLs in function also supports the idea of antigen-specific execution of CD4^+^ T cell effector functions by controlled, directional secretion of preformed effector molecules through delivery of secretory compartment to the immunological synapse [19], [20], [21]. In fact, our recent study demonstrates that pCD40L is sufficient to mediate selective activation of cognate B cells and trigger DC activation in vitro [22].

Many subsets of effector CD4^+^ T cells have been described: Th1 cells control intracellular pathogens, Th2 cells contain extracellular parasites, Th17 cells counteract extracellular bacteria and fungi, T follicular helper (T_FH_) cells promote antibody production, and regulatory T (Treg) cells prevent uncontrolled tissue damage by dampening APC activation [23]. Although other groups have reported selective expression of pCD40L in certain subsets of effector CD4^+^ T cells in disease states and healthy animals [16], [17], [24], [25], [26], [27], [28], this report is the first to systematically examine surface mobilization of pCD40L in each subset of effector CD4^+^ T cells and Treg cells, using physiologically relevant antigen-pulsed APCs to trigger surface mobilization in an effort to shed light on the role of pCD40L in vivo.

In the present study, we investigated TCR-regulated surface expression of pCD40L in Th1, Th2, Th17, and T_FH_ cells, thymocytes, invariant natural killer T (iNKT) cells and Treg cells. Our results show that pCD40L is stored in all tested subsets of effector CD4^+^ T cells from lymphoid organs and non-lymphoid effector sites as well as CD4 single positive (SP) thymocytes and iNKT cells, but is undetectable or nearly undetectable in natural or induced Treg cells, NK cells, or CD8^+^ T cells. These results provide the first comprehensive description of cells that store and mobilize pCD40L in vivo, and suggest that pCD40L may function in CD4^+^ T cell development and a broad range of immune responses in vivo.

## Materials and Methods

### Mice

Mice were housed under specific pathogen–free conditions. These studies were approved by the Institutional Animal Care and Use Committee at Oregon Health & Science University. BALB/c, C57BL/6, DO11.10, *Cd40lg^−/−^*, *β2m^−/−^*, and IL-4/enhanced GFP reporter (4get) mice were from the Jackson Laboratory (Bar Harbor, ME). DO11.10 *Rag2^−/−^* and BALB/c *nu/nu* mice were obtained from Taconic Farms (Germantown, NY). 4get/DO11.10 mice were bred in-house. SMARTA mice were obtained from Dr. J. Lindsay Whitton (The Scripps Research Institute).

### Antibodies and reagents

Biotin–anti-CXCR5 was purchased from BD Biosciences (San Jose, CA). FITC-anti-T1/ST2 was purchased from MD Biosciences (Saint Paul, MN). Allophycocyanin-labeled tetramers consisting of CD1d folded with PBS57 were provided by the NIH Tetramer Core Facility (Atlanta, GA). All other antibodies for flow cytometry were purchased from eBioscience (San Diego, CA). The Foxp3 staining kit was purchased from Biolegend (San Diego, CA). Recombinant cytokines were purchased from Peprotech (Rocky Hill, NJ). Anti-IFN-γ and anti-IL-4 were from Bio X Cell (West Lebanon, NH). Papain was from Calbiochem (San Diego, CA). Endotoxin-free ovalbumin (OVA) protein was from Profos AG (Regensburg, Germany). OVA peptide (323–339) was from AnaSpec, Inc. (Fremont, CA). All-trans retinoic acid, BSA, OVA protein, PMA and ionomycin were from Sigma-Aldrich (St Louis, MO).

### T cell differentiation in vitro

Th1, Th2, and Th17 cells were prepared by culturing spleen cells from DO11.10 mice in the presence of 1 µM of antigenic peptide (OVA 323–339) for 4 days with combinations of cytokines and antibodies as follows: Th1: 1 ng/ml IL-12 and 10 µg/ml anti-IL-4; Th2: 10 µg/ml anti-IFN-γ and 100 ng/ml IL-4; Th17: 20 µg/ml anti-IFN-γ, 20 µg/ml anti-IL-4, 20 µg/ml anti-IL-2, 100 ng/ml IL-6, 5 ng/ml human TGF-β1, 20 ng/ml IL-1β, and 20 ng/ml TNF-α. Differentiation of Th1, Th2, and Th17 cells was confirmed by intracellular cytokine staining of IFN-γ, IL-4, and IL-17A upon 5 hour stimulation with PMA plus ionomycin in the presence of brefeldin A using the Cytofix/Cytoperm kit from BD Biosciences (data not shown). In some experiments, Th2 cells were restimulated with antigen-pulsed purified B cells in the absence or presence of recombinant IL-4 or anti-IL-4 for 4 days. Stability of Th2 cells after the second round of proliferation was assessed by FACS analysis of IL-4/eGFP levels and by detection of IL-4 in the culture media with ELISA kits (BD Biosciences). To obtain induced Treg (iTreg) cells, DO11.10 cells and B cells were purified with EasySep mouse CD4^+^ T cell enrichment and B cell enrichment kits (Stemcell Technologies: Vancouver, Canada), respectively, and were co-cultured in the presence of 1 µM antigenic peptide, 100 U/ml IL-2, 20 ng/ml TGF-β, and 10 nM all-trans retinoic acid for 4 days [29].

### Lymphocytic choriomeningitis virus (LCMV) i*nfection*

To obtain in vivo-generated Th1 cells, spleen cells were prepared from recipient C57BL/6 mice that had been given 2×10^4^ spleen cells from SMARTA mice followed by i.p. infection with 2×10^5^ PFU of LCMV (Armstrong 53b strain) [17]. To assess endogenous, polyclonal Th1 and T_FH_ cells, spleen cells were harvested 12 days after LCMV infection.

### Papain plus OVA immunization

To obtain in vivo-generated Th2 and T_FH_ cells, one million 4get/DO11.10 cells (CD25-depleted, >95% purity) were transferred into BALB/c mice. The next day, recipients were immunized subcutaneously with papain (50 µg) plus endotoxin-free OVA protein (50 µg) [30]. On day 7, Th2 and T_FH_ cells from draining lymph nodes (dLNs) were prepared for flow cytometric analysis.

### Asthma model

To obtain polyclonal Th2 cells, C57BL/6 mice were sensitized twice by i.p. injection of OVA protein emulsified in aluminum hydroxide (Pierce, Rockford, IL) and challenged intratracheally with OVA protein/PBS three times. Mice were sacrificed, tracheas were cannulated, and lungs were lavaged three times with 0.5 ml PBS per wash to obtain bronchoalveolar lavage fluid (BALF) cells [31].

### Experimental autoimmune encephalomyelitis (EAE) model

For analysis of in vivo-generated Th17 cells and Treg cells from an inflammatory site, active EAE was induced and CNS infiltrating leukocytes were obtained as described [32]. Briefly, C57BL/6 mice were immunized by subcutaneous injection in the lower back with 200 µg myelin oligodendrocyte glycoprotein (MOG)35–55 (MEVGWYRSPFSRVVHLYRNGK) peptide emulsified at a 1∶1 ratio with complete Freund's adjuvant containing 150 µg *Mycobacterium tuberculosis* H37RA (Difco, Detroit, MI). Pertussis toxin (List Biological Laboratories, Campbell CA) was administered on day 0 (200 ng) and day +2 (200 ng) with respect to the immunization day. Only symptomatic mice were used at 14 days after immunization.

### Oral tolerance model

In vivo-generated iTreg cells were prepared from mesenteric LNs of BALB/c *nu/nu* recipients which had received i.v. injection of 5×10^5^ naive CD4^+^ T cells purified from DO11.10 *Rag2^−/−^* mice followed by feeding with BSA- or OVA-containing water for 6 days. [33].

### Flow cytometry for detection of pCD40L

The surface mobilization assay and intracellular staining were described previously [17] and are explained at the beginning of the Results section. To detect pCD40L in Th17 cells generated in vivo, CNS infiltrating leukocytes from EAE animals were analyzed by the CD40L mobilization assay followed by intracellular cytokine staining. Cells were incubated in the presence or absence of MOG peptide-pulsed APCs with either isotype-PE or PE-labeled anti-CD40L at 37°C for 30 minutes. After washes, cells were incubated for 4.5 hours in the presence of brefeldin A and MOG peptide-pulsed APCs. After fixation, intracellular IL-17A and IFN-γ were stained. Data were obtained with an LSR II (BD Biosciences) and analyzed with FlowJo software (Tree Star, Inc., Ashland, OR).

## Results

### Detecting pCD40L using the mobilization assay and intracellular staining

We previously reported successful use of the mobilization assay and intracellular staining to assess existence of pCD40L in Th1 effector and memory cells as well as memory-phenotype CD4^+^ T cells [17], [22]. It has been reported that CD40 engagement induces CD40L internalization [34] and that inhibition of CD40-CD40L engagement with blocking anti-CD40L increases CD40L detection [35]. In the mobilization assay, fluorochrome-labeled anti-CD40L mAb is included in the culture at 37°C in the presence or absence of stimulation. Compared to the “snap shot” nature of conventional staining at 4°C after completion of stimulation, the mobilization assay provides the “long exposure” view of CD40L surface expression by capturing and stabilizing CD40L that has been delivered to the cell surface during incubation (Fig. S1) [36], [37]. We found negligible amounts of pre-existing surface CD40L on resting effector CD4^+^ T cells [17]. By limiting the stimulation period to 30 minutes, we were able to exclude surface expression of de-novo CD40L made following stimulation. Although a contribution of new CD40L protein expression from stable pre-existing CD40L mRNA cannot be fully excluded by limiting the assay to 30 minutes [38], we showed in a previous report with T effectors that complete inhibition of protein synthesis did not diminish surface expression of CD40L following 30 minutes of stimulation [17]. Intracellular staining can be seen as an “x-ray”, and could distinguish defective mobilization of pCD40L from absence of stored pCD40L in cases where no mobilization of pCD40L is observed.

### In vitro-generated Th1 and Th17, but not Th2 or iTreg cells, store and mobilize pCD40L upon stimulation

We previously showed that effector and resting memory Th1 cells, differentiated either in vitro or in vivo, store intracellular pCD40L and mobilize it to the cell surface within 30 minutes of stimulation [17]. A finding that pCD40L is stored in certain Th subsets would suggest that pCD40L serves in defined immune responses, just as signature cytokines do. Therefore, we examined the distribution of pCD40L among subsets of effector CD4^+^ T cells and Treg cells.

**Figure 1:**
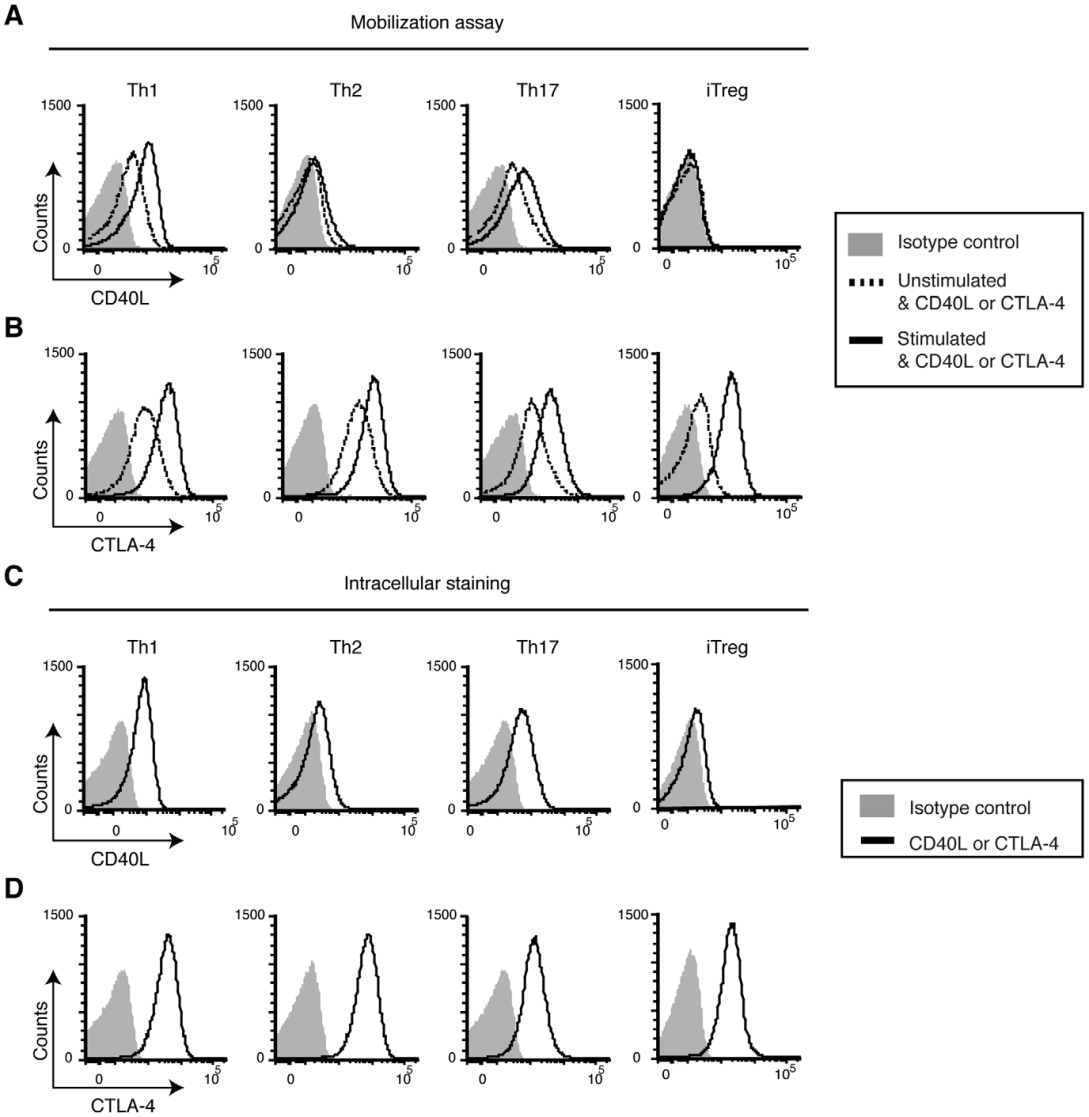
In vitro-generated Th1 and Th17, but not Th2 or iTreg cells mobilize pCD40L. *A* and *B*, Mobilization assay. In vitro-generated Th1, Th2, Th17, and iTreg cells were stimulated with PMA plus ionomycin or left unstimulated in the presence of PE-isotype Ab, PE-anti-CD40L or PE-anti-CTLA-4 at 37°C for 30 minutes. The levels of CD40L (*A*) and CTLA-4 (*B*) are shown. *C* and *D*, Intracellular staining. Cells were fixed without stimulation, permeabilized, and stained with PE-isotype Ab, PE-anti-CD40L or PE-anti-CTLA-4. The levels of CD40L (*C*) and CTLA-4 (*D*) are shown. Data are representative of five independent experiments.

We generated Th1, Th2, Th17, and iTreg cells in vitro and measured pCD40L. While Th1 and Th17 cells clearly mobilize pCD40L, Th2 cells mobilize much less pCD40L (Fig. 1*A*) and possess significantly less intracellular CD40L (Fig. 1*C*). pCD40L is at the limit of detection in iTreg cells (Figs. 1*A, 1C*). The decreased surface mobilization in Th2 cells, and the near absence of pCD40L in Treg cells, are not due to suboptimal stimulation or permeabilization of these cells because Th2 and iTreg cells possess and mobilize preformed CTLA-4 at the same level as Th1 and Th17 cells (Figs. 1*B, 1D*).

### In vivo-generated Th1 and T_FH_ cells possess and mobilize pCD40L upon antigenic stimulation

**Figure 2:**
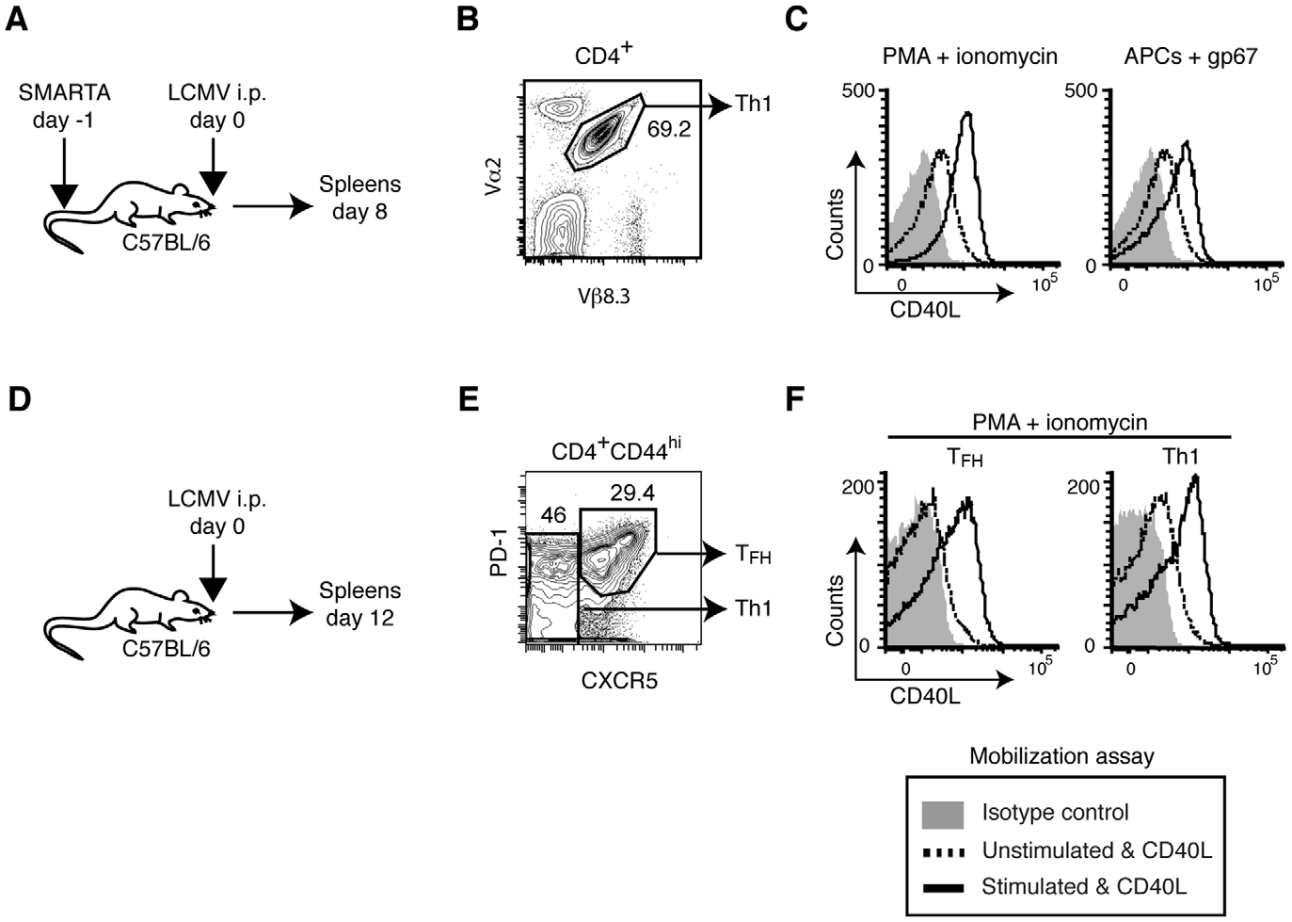
In vivo-generated Th1 and T_FH_ cells possess and mobilize pCD40L. *A*, Generation of antigen-specific Th1 cells. LCMV-glycoprotein-specific, TCR-transgenic SMARTA CD4^+^ T cells were recovered from LCMV-infected mice on day 8 post-infection. *B*, Gating strategy for SMARTA cells (Vα2^+^, Vβ8.3^+^). *C*, Mobilization of pCD40L by SMARTA cells in response to PMA plus ionomycin or the LCMV peptide gp67-pulsed APC. *D*, Polyclonal Th1 and T_FH_ cells. Spleen cells were obtained from LCMV-infected mice on day 12 post-infection. *E*, Gating strategy for T_FH_ (CD4^+^CD44^hi^CXCR5^hi^PD-1^hi^) and Th1 (CD4^+^CD44^hi^CXCR5^low^PD-1^low^) cells. *F*, Mobilization of pCD40L by T_FH_ and Th1 cells in response to PMA plus ionomycin. Data are representative of two independent experiments.

To further characterize the involvement of pCD40L in vivo, we examined effector CD4^+^ T cell subsets generated in vivo. SMARTA CD4^+^ T cells, which have a transgenic TCR specific for an LCMV epitope [39], were transferred into normal mice followed by infection with LCMV (Fig. 2*A*). Th1 differentiation of these cells was confirmed by intracellular staining of IFN-γ upon in vitro stimulation with PMA plus ionomycin (60–70% IFN-γ^+^, data not shown). The mobilization assay shows that in vivo-generated Th1 SMARTA (CD4^+^Vα2^+^Vβ8.3^+^, Fig. 2*B*) cells mobilize pCD40L in an antigen-specific manner (Fig. 2*C*). SMARTA cells expanded during LCMV infection were reported to contain both Th1 and T_FH_ cells [40]. To rule out the possibility of preferential pCD40L expression in T_FH_ cells rather than Th1 cells, mobilization of pCD40L was assessed in endogenous polyclonal T_FH_ cells (CD4^+^CD44^hi^CXCR5^hi^PD-1^hi^) and Th1 cells (CD4^+^CD44^hi^CXCR5^low^PD-1^low^) from spleens of LCMV-infected mice (Figs. 2*D, 2E*). The result shows that both Th1 and T_FH_ cells mobilize pCD40L upon stimulation (Fig. 2*F*).

### In vivo-generated Th2 cells possess and mobilize pCD40L upon antigenic stimulation

**Figure 3:**
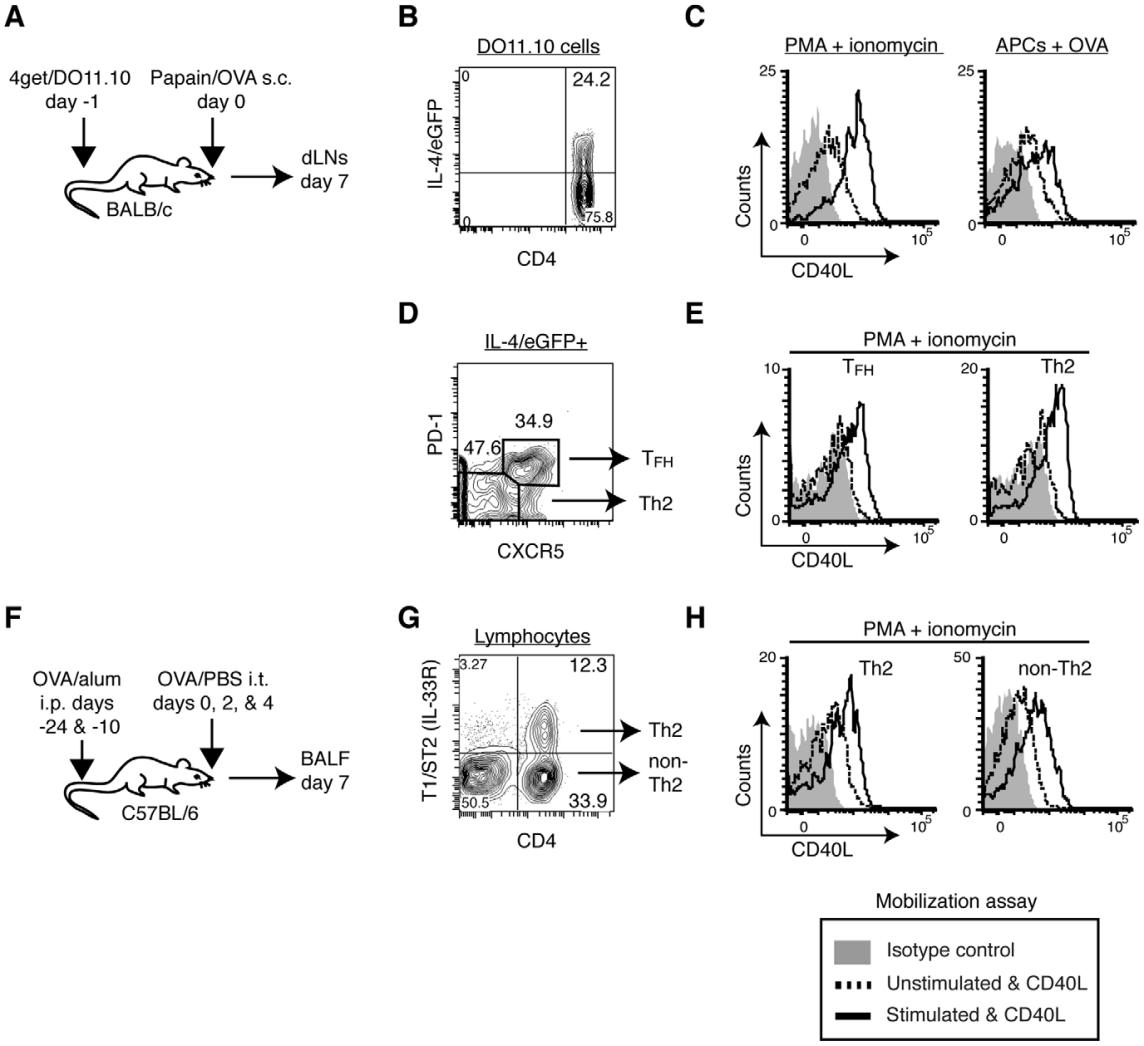
In vivo-generated Th2 cells store and mobilize pCD40L. *A*, BALB/c mice that had received 4get/DO11.10 CD4^+^ T cells were subcutaneously immunized with papain plus OVA protein. Seven days later, cells from dLNs were analyzed by the mobilization assay. *B*, Gating strategy for antigen-specific IL-4/eGFP^+^ DO11.10 cells (CD4^+^IL-4/eGFP^+^KJ1-26^+^). *C*, Mobilization of pCD40L by IL-4/eGFP^+^ DO11.10 cells in response to PMA plus ionomycin or OVA peptide-pulsed APC. *D*, Those cells were further differentiated as T_FH_ (CXCR5^hi^PD-1^hi^) and Th2 (CXCR5^low^PD-1^low^) cells. *E*, Mobilization of pCD40L by T_FH_ and Th2 cells in response to PMA plus ionomycin. *F*, To obtain polyclonal Th2 cells, mice were sensitized with OVA/alum followed by intratracheal challenge with OVA/PBS. *G*, The gating strategy for Th2 cells (CD4^+^T1/ST2^+^) and non-Th2 cells in BALF. *H*, Mobilization of pCD40L by polyclonal Th2 and non-Th2 cells in response to PMA plus ionomycin. Data are representative of two to three independent experiments.

**Figure 4:**
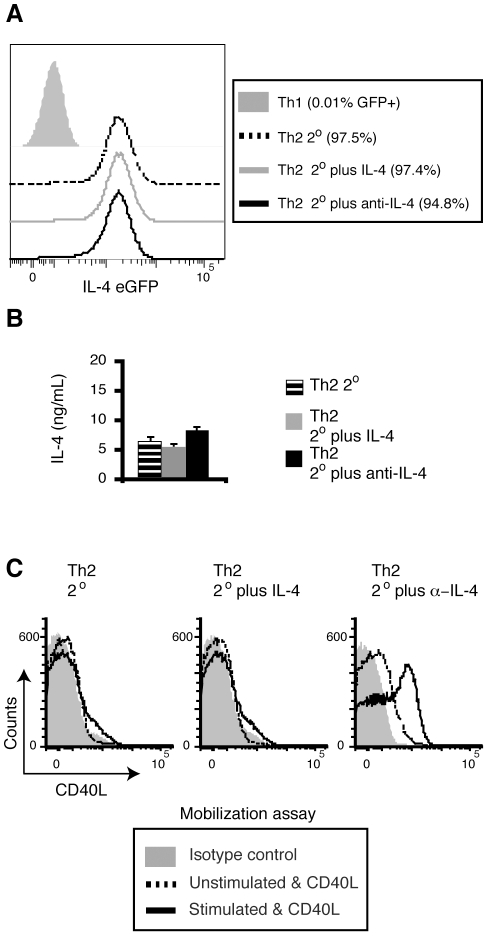
Excess IL-4 downregulates pCD40L levels in Th2 cells generated in vitro. Recovery of pCD40L in Th2 cells by neutralizing IL-4. *A–C*, Differentiated Th2 cells from 4get/DO11.10 spleen cells were restimulated with peptide-pulsed splenic APCs alone (Th2 2°) or in the presence of exogenous IL-4 (Th2 2° plus IL-4) or anti-IL-4 (Th2 2° plus anti-IL-4) for 4 days. *A*, The levels of IL-4/eGFP in each group of Th2 cells as well as Th1 negative control cells. *B*, IL-4 production upon PMA plus ionomycin stimulation by each group of Th2 cells was analyzed by ELISA. Each bar represents the mean ± standard deviation for triplicates. *C*, Mobilization of pCD40L by each group of Th2 cells in response to PMA plus ionomycin. Data are representative of two independent experiments.

Next, we tested in vivo-generated Th2 cells by transferring CD4^+^ T cells from 4get/DO11.10 TCR transgenic mice into normal mice and immunizing recipients with papain plus OVA protein, which induces a robust Th2 response in vivo [30] (Fig. 3*A*). A week after immunization, 20 to 40% of the DO11.10 CD4^+^ T cells became IL-4/eGFP positive Th2 cells (Fig. 3*B* and data not shown). In contrast to what we observed with in vitro-generated Th2 cells, in vivo-generated Th2 cells mobilize a substantial amount of pCD40L upon cognate interactions with APCs (Fig. 3*C*). To rule out the possibility of preferential pCD40L expression in T_FH_ cells rather than in Th2 cells, we conducted the mobilization assay for pCD40L followed by staining of CXCR5 and PD-1 [26], [41]. The mobilization assay shows that both T_FH_ (IL-4/eGFP^+^CXCR5^hi^PD-1^hi^) and Th2 (IL-4/eGFP^+^CXCR5^low^PD-1^low^) DO11.10 cells mobilize pCD40L (Figs. 3*D, 3E*). To further verify these findings, we analyzed endogenous, polyclonal Th2 cells in an asthma model (Fig. 3*F*). We observed mobilization of pCD40L in polyclonal Th2 cells, which were identified by a Th2 marker (T1/ST2, IL-33R), in BALF from sensitized and challenged animals (Figs. 3*G, 3H*) [42]. These results were surprising since in vitro-generated Th2 cells stored and mobilized very little pCD40L. To determine the root of this discrepancy, we considered cell-extrinsic factors that might downregulate pCD40L in Th2 cells generated in vitro. Because IL-4 has been shown to repress the late phase of de novo CD40L expression by stimulated naïve CD4^+^ T cells [43], we tested whether exogenous or accumulated IL-4 in the culture is responsible for the diminished pCD40L expression in Th2 cells. In vitro-generated Th2 cells were prepared as in Fig. 1 and then restimulated with antigen-pulsed APCs either in the absence or presence of recombinant IL-4 or neutralizing anti-IL-4 for 4 days. After the two rounds of stimulation, all groups of Th2 cells maintained their stable Th2 phenotype as measured by IL-4/eGFP expression (Fig. 4*A*) and IL-4 protein secretion upon PMA plus ionomycin stimulation (Fig. 4*B*). When the three groups of Th2 cells were analyzed, we found that only Th2 cells that underwent the second round of stimulation in the presence of anti-IL-4 mobilized pCD40L upon stimulation (Fig. 4*C*). These data indicate that a non-physiological level of IL-4 in the in vitro culture causes downregulation of pCD40L. IL-4 levels in situ in lymph nodes undergoing an intense Th2 response were estimated to be 15–500 pg/ml [44], whereas the culture media in this experiment may contain 5–100 ng/ml of IL-4. We conclude that Th2 cells do store and mobilize pCD40L except in the presence of high levels of exogenous or accumulated IL-4.

### In vivo-generated Th17 cells possess and mobilize pCD40L upon antigenic stimulation

**Figure 5:**
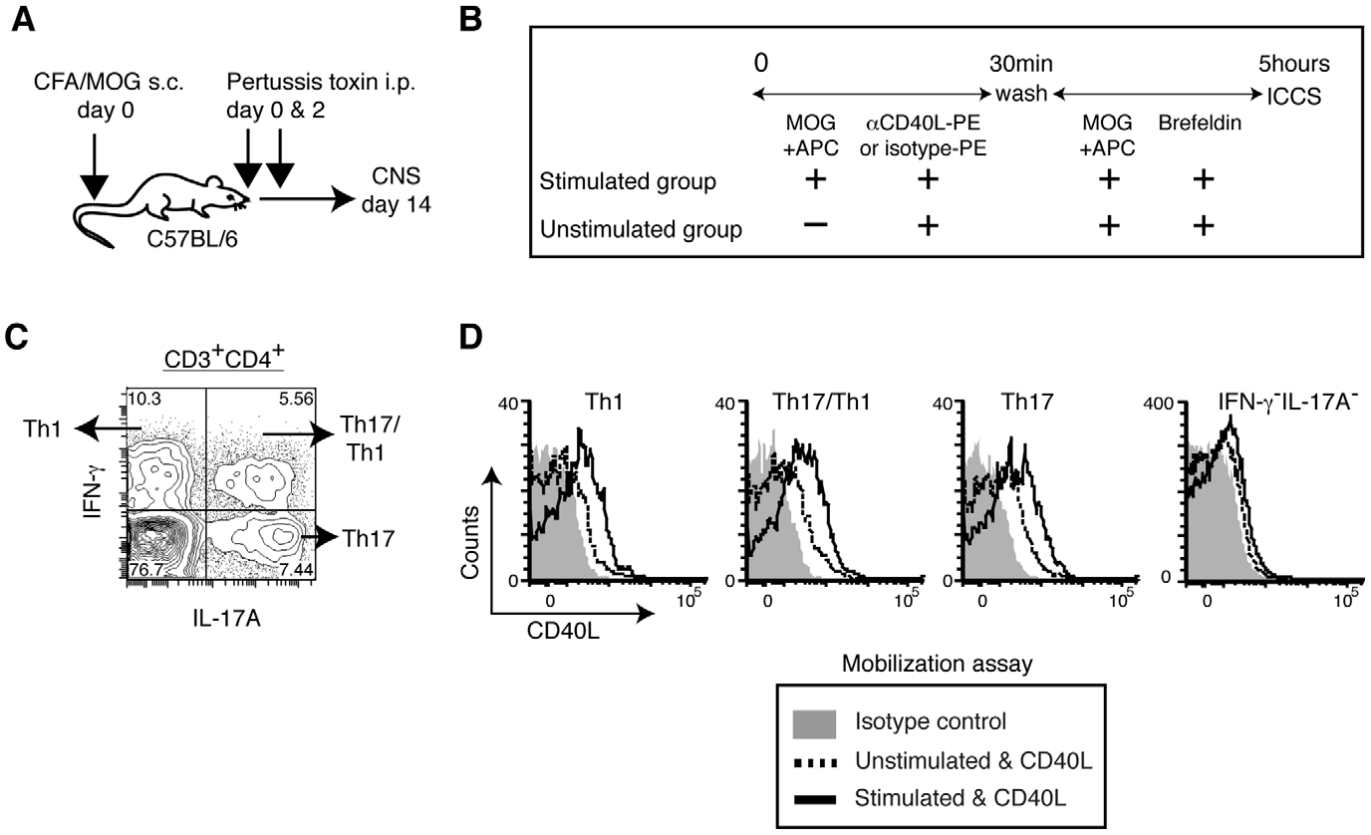
In vivo-generated Th17 and Th17/Th1 cells store and mobilize pCD40L. *A*, CNS infiltrating leukocytes were obtained from brains and spinal cords of EAE animals induced by immunization with CFA plus MOG peptide and pertussis toxin. *B*, CNS infiltrating leukocytes were analyzed by the CD40L mobilization assay followed by intracellular cytokine staining. *C*, Intracellular staining of IL-17A and IFN-γ in CNS CD4^+^ T cells. *D*, CD40L mobilization in Th1 (IL-17A^−^IFN-γ^+^), Th17 (IL-17A^+^IFN-γ^−^), Th17/Th1 (IL-17A^+^IFN-γ^+^), and antigen non-specific (IL-17A^−^IFN-γ^−^) CD4^+^ T cells upon antigenic stimulation. Data are representative of three independent experiments.

We also tested in vivo-generated Th17 cells for pCD40L using an EAE model (Fig. 5*A*). To distinguish Th17 cells from Th17/Th1 and Th1 cells in the mobilization assay, cells from the CNS of symptomatic EAE animals were analyzed with a combination of intracellular cytokine staining and the mobilization assay (Fig. 5*B*). Endogenous Th17 cells, as well as Th17/Th1 and Th1 cells, but not antigen non-specific (IFN-γ^−^IL-17A^−^) CD4^+^ T cells from EAE lesions mobilize pCD40L upon antigen recognition (Figs. 5*C*, 5D). Together, the findings above show that pCD40L is widely shared among effector CD4^+^ T cells.

### Treg cells possess little or no pCD40L

**Figure 6:**
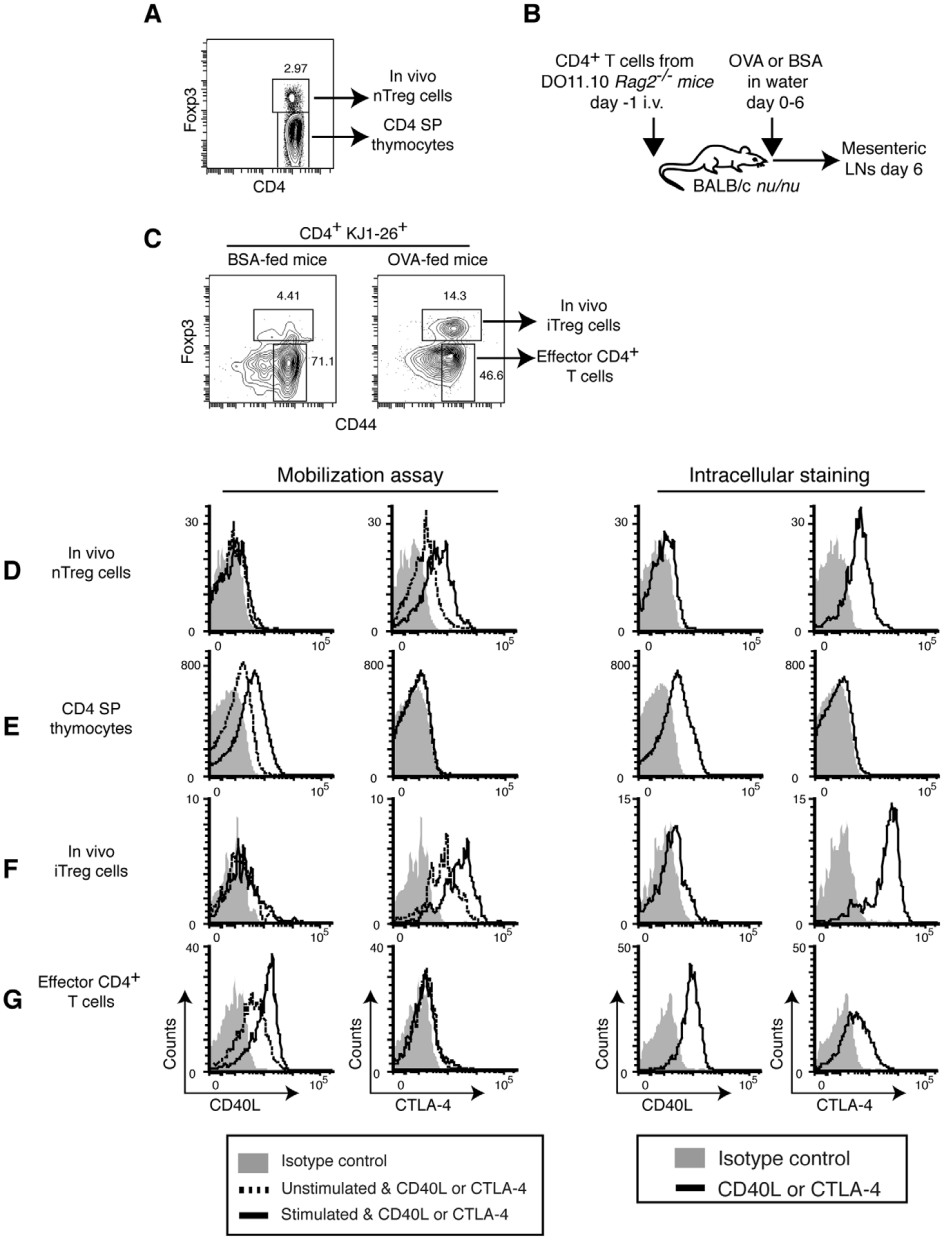
CD4 SP thymocytes, but not Treg cells, store and mobilize pCD40L. *A*, Gating strategy for thymic nTreg cells and CD4 SP thymocytes. *B*, Generation of in vivo iTreg cells. DO11.10 CD4^+^ T cells were recovered from OVA- or BSA-fed recipient mice on day 6. *C*, Gating strategy for in vivo iTreg cells and effector CD4^+^ T cells. A BSA-fed mouse is shown as a negative control. *D*, thymic nTreg cells; *E*, CD4 SP thymocytes; *F*, in vivo iTreg cells; and *G*, effector CD4^+^ T cells, are analyzed by the mobilization assay and intracellular staining for pCD40L and CTLA-4. Data are representative of three (*D* and *E*) or two (*F* and *G*) independent experiments.

Although Treg cells are reported to accumulate low levels of surface CD40L in the steady state in *CD40^−/−^* mice [45], we observed little or no pCD40L in iTreg cells generated in vitro (Fig. 1). We observed differences in pCD40L level between in vitro and in vivo Th2 cells (Figs. 1, 3, and 4), leading us to examine pCD40L expression and mobilization in Treg cells obtained in vivo. We were unable to detect unambiguous pCD40L expression in either thymic natural Treg (nTreg) cells or splenic Treg cells from unmanipulated mice, although they both express and mobilize CTLA-4 (Figs. 6*A, 6D* and Figs. S2 *A*, S2*B*). In accord with this notion, exclusion of CD44^hi^ nTreg cells from memory phenotype (MP) CD4^+^ T cells resulted in higher pCD40L mobilization than previously reported [17] (Figs. S2 *A*, S2*C*). Although we previously concluded that naïve CD4^+^ T cells do not have pCD40L [17], we reproducibly detected a very low level of pCD40L in naïve CD4^+^ T cells (Figs. S2 *A*, S2*D*). Low level pCD40L expression in naïve CD4^+^ T cells has also been reported by others [28].

**Figure 7:**
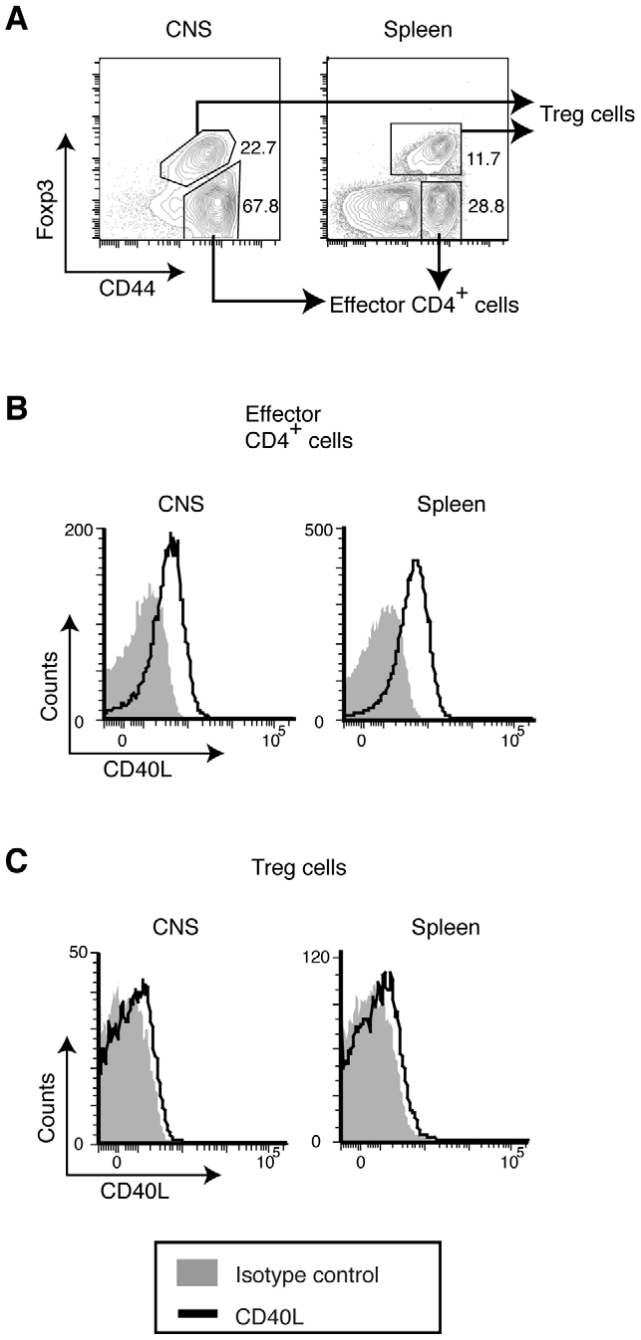
Severe inflammation does not compromise the lack of expression of pCD40L by Treg cells. CNS infiltrating leukocytes and splenocytes were obtained from EAE animals induced as Fig. 5*A*. *A*, Gating strategy for cells from CNS and spleen. *B & C*, Intracellular CD40L levels for effector CD4^+^ T cells (*B*) and Treg cells (*C*). Data are representative of two independent experiments.

Next, we tested antigen-driven, in vivo-generated iTreg cells induced in an oral tolerance model with OVA [33] (Figs. 6*B, 6C*). Although it has been shown that CD40L is required for the induction of oral tolerance [46], we found that in vivo-generated iTreg cells nearly lack pCD40L expression (Fig. 6*F*), while the CD44^hi^ effector CD4^+^ T cells in the same model had a high level of pCD40L (Fig. 6*G*). Because this finding was obtained in a tolerogenic environment, we next examined whether Treg cells might acquire pCD40L in an inflammatory environment. In EAE, most Treg cells in inflamed CNS tissue are nTreg cells of thymic origin [47]. Even though almost all Treg cells in the CNS upregulate CD44 (Fig. 7*A*) and effector CD4^+^ T cells acquire abundant pCD40L (Fig. 7*B*), Treg cells from inflamed CNS as well as spleens lacked detectable pCD40L (Fig. 7*C*). Taken together, these data indicate that pCD40L expression in Treg cells is tightly suppressed.

### CD4 SP thymocytes and iNKT cells possess pCD40L

**Figure 8:**
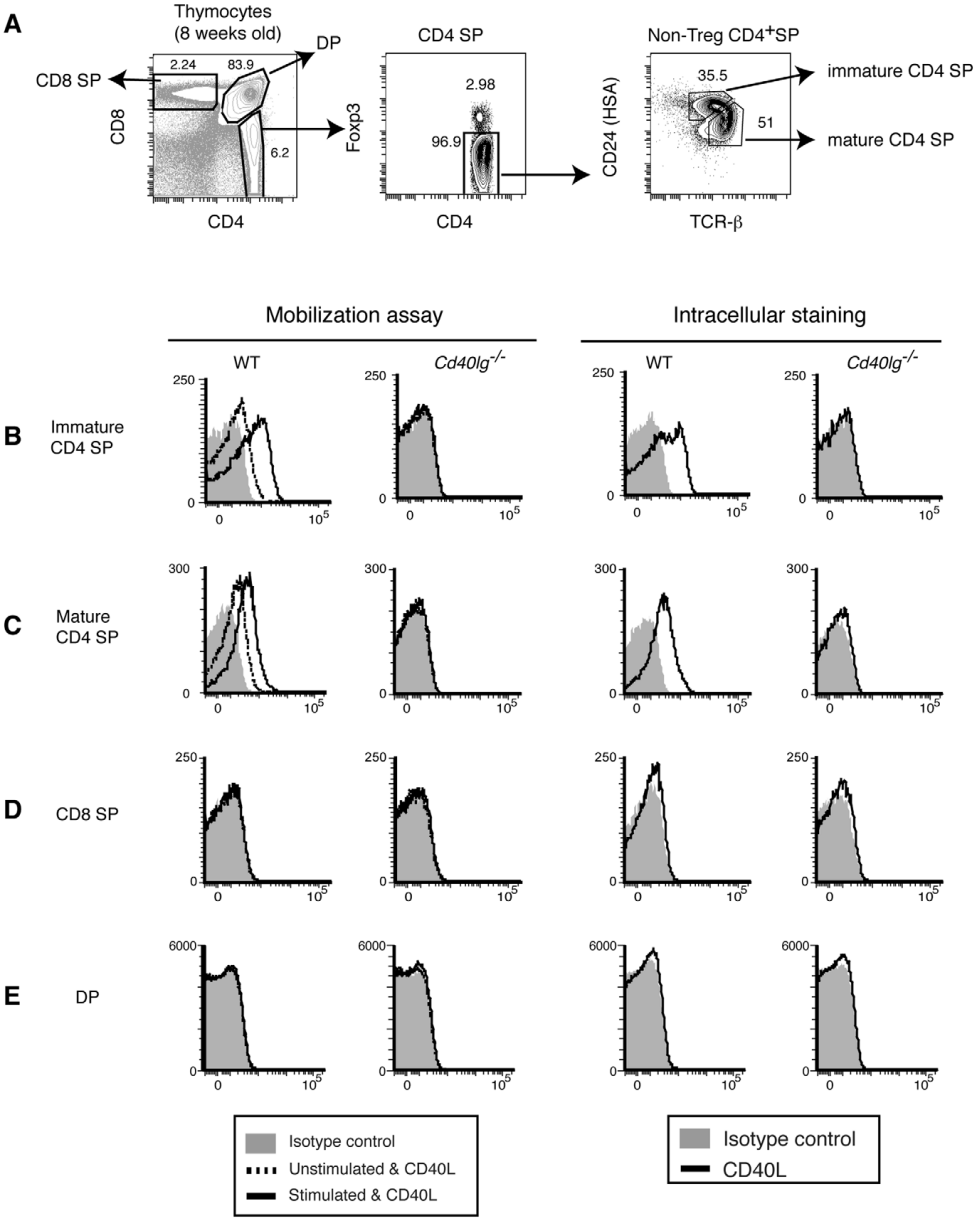
CD4 SP thymocytes, but not CD8 SP or DP thymocytes, store and mobilize pCD40L. *A*, Gating strategy for identifying CD8 SP, DP, and immature and mature CD4 SP thymocytes. *B–E*, Mobilization of pCD40L for immature (*B*) and mature (*C*) CD4 SP thymocytes, CD8 SP (*D*), and DP (*E*) thymocytes. *Cd40lg^−/−^* : CD40L-deficient mouse. Data are representative of at least five independent experiments.

We were surprised to see low level pCD40L expression in peripheral naïve CD4^+^ T cells. This suggested to us that there might be a developmental role for pCD40L, so we decided to examine pCD40L in thymic T cell populations. When we looked in the thymus, we found that CD4 SP (CD4^+^CD8^−^Foxp3^−^) thymocytes express and mobilize pCD40L (Fig. 6*E*). Further analysis showed that immature CD4 SP thymocytes have more pCD40L than mature CD4 SP thymocytes (Figs. 8*A, B, C*). We could not detect any CD40L staining in CD4^−^CD8^+^ (CD8 SP) and CD4^+^CD8^+^ double-positive (DP) thymocytes (Figs. 8*D, 8E*). These results suggest that immature CD4 SP thymocytes acquire pCD40L, and then decrease pCD40L to lower levels as they mature.

**Figure 9:**
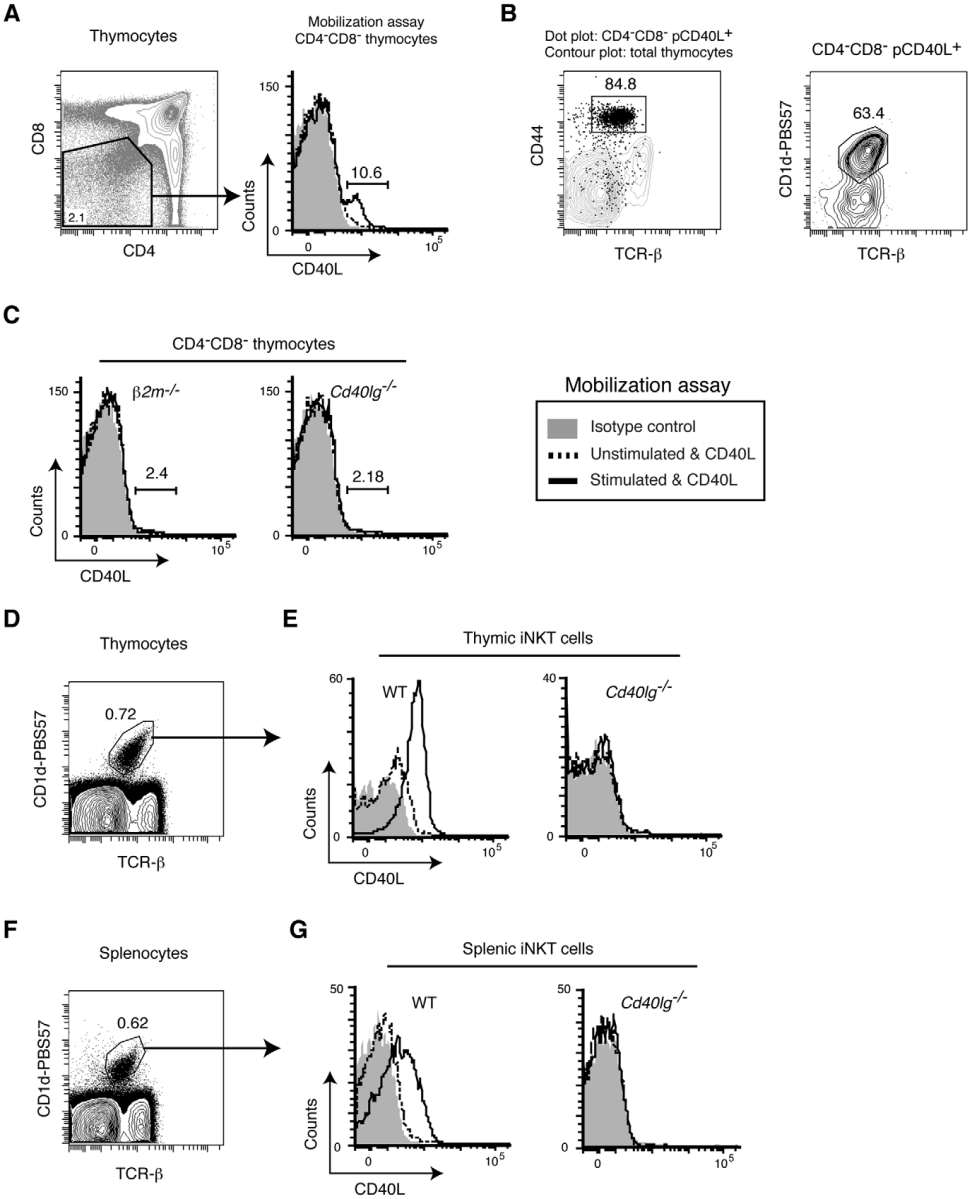
iNKT cells possess and mobilize pCD40L. *A*, A fraction of the DN thymocyte population mobilizes pCD40L upon stimulation with PMA plus ionomycin. *B*, pCD40L positive DN thymocytes are iNKT cells. *C*, iNKT deficient *β2m−/−* mice lack the pCD40L positive DN thymocyte population. *D and F*, Gating strategy for thymic (D) and splenic (F) iNKT cells. *E and G*, Mobilization of pCD40L in CD1d-tetramer-positive thymic (*E*) or splenic (*G*) iNKT cells upon stimulation with PMA plus ionomycin. Data are representative of two independent experiments.

We also detected mobilization of pCD40L in a small fraction of the CD4^−^CD8^−^ double-negative (DN) population (Fig. 9*A*). Further analysis showed that those cells were CD44^hi^, TCR-β^int^, and CD1d-PBS57 tetramer positive iNKT cells (Fig. 9*B*). In fact, in β2m-deficient (*β2m^−/−^*) mice, which lack both CD8^+^ T cells and iNKT cells, the pCD40L positive population in DN thymocytes is absent (Fig. 9*C*). Direct assessment of thymic iNKT cells shows clear and uniform mobilization of pCD40L (Figs. 9*D, 9E*). Splenic iNKT had somewhat reduced pCD40L (Figs. 9*F, 9G*). We found no detectable pCD40L in NK cells and CD8^+^ T cells (Figs. S3 *A*, S3*B*) [17], [48].

## Discussion

Recent two-photon microscopy studies indicate that interactions between effector CD4^+^ T cells and APCs in vivo are surprisingly brief [12], [13], [14], [15], necessitating a reassessment of our ideas about how CD4^+^ T cells deliver their effector functions. The dominant idea is directional secretion of newly synthesized cytokines toward antigen-bearing APCs [49], but de novo cytokine synthesis requires more time than is provided by these short in vivo interactions. On the other hand, preformed effector molecules stored in T cells can be delivered by regulated secretion in minutes as opposed to hours. As a membrane-bound cytokine, CD40L is designed for delivery by cell contact, and is necessary for cognate help for B cells and licensing of DC [1], [2], so regulated surface expression of pCD40L could explain antigen-specific activation of APC in brief interactions with CD4^+^ T cells in vivo. pCD40L has been reported in CD4^+^ T cells [16], [17], [24], [25], [26], [27], [28], but it is not yet known which functions of CD40L require de novo CD40L synthesis and which can be supplied by pCD40L. Our recent study showed that pCD40L is sufficient for the activation of DCs and selective activation of antigen-bearing B cells in vitro [22]. In this paper, we assessed possession and surface mobilization of pCD40L among the newly expanded classification of CD4^+^ T cell subsets by signature cytokines [23] to determine whether pCD40L is restricted to specific subsets with certain functions, for example, T_FH_ or Th1 cells. We found instead that pCD40L is expressed and mobilized to the cell surface in all tested effector CD4^+^ T cell subsets, as well as CD4 SP thymocytes and iNKT cells. However, neither nTreg nor iTreg cells possess easily detectable pCD40L. Taken together, our recent findings indicate that pCD40L may be involved in T cell development and function broadly in innate and adaptive immunity.

pCD40L may also contribute to the pathology of inflammatory diseases because pCD40L is not limited to the primary and secondary lymphoid organs. Th2 cells recovered from the airway of a mouse asthma model and Th1 and Th17 cells from CNS disease lesions of EAE animals possess and mobilize pCD40L. Similar findings were reported in effector CD4^+^ T cells recovered from synovial fluid of human rheumatoid arthritis patients [24]. It has been shown that anti-CD40L treatment not only blocks the induction phase but also ameliorates the effector phase of EAE [50], [51]. Two-photon microscopy shows predominantly brief interactions of effector memory CD4^+^ T cells with APCs in target tissues [14]. Together, these findings suggest that pCD40L may function during the effector phase of inflammation through the promotion of cytokine secretion by APCs.

Strikingly, we found that only Treg cells lack reproducibly detectable pCD40L among all CD4^+^ T cells, further suggesting that activation of APCs is the primary role of pCD40L. In contrast, it has been reported that a fraction of Treg cells express de novo CD40L upon activation [52] and elicit CD8 T cell responses in a CD40L-dependent manner in vivo [53]. These cases can be seen as functional reprogramming of Treg cells to manifest helper/effector activity [54]. Therefore, one might imagine that aberrant pCD40L expression in Treg cells could be observed in severe inflammation. However, Treg cells defined by Foxp3 expression had barely detectable pCD40L expression even in the presence of pathologic inflammation caused by Th1 and Th17 cells in EAE lesions, in keeping with a recent report that showed an extremely stable phenotype of Treg cells [55]. Alternatively, ex-Treg cells that have lost Foxp3 expression might gain pCD40L after conversion to aggressive effector CD4^+^ T cells [56]. In fact, converted ex-nTreg cells in the gut preferentially become T_FH_ cells and express CD40L [57]. Based on the above findings, it would be interesting to test whether engineering Treg cell-specific expression of pCD40L could redirect their activity from regulation to activation of APCs.

There are several non-mutually exclusive mechanisms that could explain the lack of pCD40L in Treg cells. One possibility is transcriptional repression. It was shown that Foxp3 can form a heterodimer with NFAT1, and that the NFAT1:Foxp3 complex prevents the NFAT1:AP-1 complex from binding to the IL-2 promoter, resulting in repression of IL-2 mRNA transcription [58]. The CD40L promoter has a consensus sequence (5′-**GGAA**NNNN**TGTTT**-3′) for the NFAT1:Foxp3 complex [58], suggesting repression of CD40L mRNA transcription by the NFAT1:Foxp3 complex. In fact, a reduced level of CD40L mRNA was found in Treg cells compared to naïve CD4^+^ T cells [45]. CD4^+^ T cells from Foxp3 transgenic mice also showed reduced CD40L expression upon anti-CD3 plus anti-CD28 stimulation [59]. Independent of Foxp3, anergic CD4^+^ T cells produced by TCR stimulation without costimulation, presumably through NFAT in the absence of AP-1 [60], also lack CD40L expression [61], [62]. Our preliminary data indicate that type 1 regulatory T (Tr1) cells [63] lack pCD40L, strengthening the link between diminished pCD40L and an anergic phenotype (Y. Koguchi, D.C. Parker, unpublished data). Post-transcriptional regulation of CD40L mRNA may be different between effector CD4^+^ T cells and Treg cells because CD40L mRNA is stabilized in response to Ag recognition in effector T cells [64]. Another possibility is post-translational modification of pCD40L. It is reported that GRAIL (gene related to anergy in lymphocytes) directly downregulates CD40L through its E3 ubiquitin ligase activity [65], although this finding is controversial since GRAIL-deficient mice from two different groups did not show any evidence of CD40L overexpression [66], [67]. Investigation of the cause(s) of the lack of pCD40L in Treg cells could shed light on how effector CD4^+^ T cells acquire and regulate pCD40L.

The selective expression of pCD40L in CD4 SP thymocytes implies a role in T cell development. Through a careful examination of pCD40L in thymocytes, we found that CD4 SP thymocytes have pCD40L and immature CD4 SP thymocytes have more pCD40L than mature CD4 SP thymocytes. CD40L is necessary for thymic negative selection to endogenous superantigens [68] and contributes to the maintenance of medullary thymic epithelial cells (mTECs) [69], [70], [71]. Unregulated expression of surface CD40L on thymocytes in CD40L transgenic mice caused hyper-proliferation of mTECs [6]. Therefore, it seems likely that regulated provision of pCD40L by CD4 SP thymocytes plays an important role in homeostasis of mTECs. Provision of pCD40L by CD4 SP thymocytes may also be important for homeostasis of Treg cells by promoting IL-2 production from DCs [72].

iNKT cells, as innate immune cells, produce proinflammatory and immunoregulatory cytokines and deliver effector functions that depend on perforin and granzyme B [73]. Although de novo CD40L expression in iNKT cells has been reported [74], [75], [76], this is the first report of the presence of pCD40L in iNKT cells. The contribution of CD40L to iNKT cell development has not been studied. Although it is still a matter of debate, CD1d expressing thymic DCs may mediate negative selection of iNKT cells [77], in which case, pCD40L may play a role in that process. In the periphery, iNKT cells can mediate acute hepatitis in response to Con A in a CD40L-dependent manner [78], and provide cognate help for iNKT glycolipid-pulsed B cells via delivery of CD40L [79], [80]. iNKT cells can license DCs and myeloid-derived suppressor cells via delivery of CD40L for optimal CD4^+^ and CD8^+^ T cell responses [81], [82], [83]. A recent study using two-photon microscopy showed that cognate interactions between lymph node macrophages and iNKT cells are relatively stable (only 20% show short interaction less than 20 min) [84] allowing ample time for de novo CD40L production. Whether cognate interactions of iNKT cells with DCs and B cells are stable or transient is currently unknown. Further studies are required to address whether iNKT cells mobilize pCD40L upon stimulation with glycolipid-pulsed APCs and whether any of the CD40L-dependent functions of iNKT cells are owing to pCD40L.

The ability of pCD40L to be rapidly mobilized to the cell surface upon antigen recognition provides a mechanism for T cells to activate cognate APC during transient, antigen-specific interactions in vivo. The broad distribution of pCD40L among CD4^+^ T effector cell subsets and iNKT cells indicates that use of pCD40L may be widespread in T cell development and innate and adaptive immune responses. Future research to define the molecular machinery that regulates the formation of the pCD40L secretory compartment and its delivery to the cell surface will allow studies in vivo to determine which of the many known functions of CD40L are owing to pCD40L, and provide new targets for therapeutic intervention in inflammatory diseases.

## Supporting Information

Figure S1 **Schematic explanation of the mobilization assay.** In the mobilization assay, fluorochrome-labeled anti-CD40L mAb is included in the culture during the activation of cells at 37°C. Compared to the “snap shot” nature of conventional staining at 4°C after completion of stimulation, the mobilization assay captures CD40L that has been delivered to the cell surface during stimulation while blocking CD40-dependent internalization, thereby providing the “long exposure” view of CD40L surface expression. By limiting the stimulation period to 30 minutes, we were able to exclude surface expression of de-novo CD40L made following stimulation (Koguchi, 2007). Intracellular staining can be seen as an “x-ray”, and is useful to distinguish defective mobilization of pCD40L from absence of stored pCD40L in cases where no mobilization of pCD40L is observed.
(TIF)

Figure S2 **pCD40L expression in splenic CD4+ T cell subsets.** *A*, Gating strategy for Treg cells, memory phenotype (MP) and naive CD4^+^ T cells. *B–D*, Mobilization of pCD40L upon stimulation with PMA plus ionomycin and intracellular staining of Treg cells (*B*), MP (*C*) and naive (*D*) CD4^+^ T cells. Data are representative of seven independent experiments.
(TIF)

Figure S3 **NK cells and CD8^+^ T cells do not have pCD40L.** Gating strategies and the data for the mobilization of pCD40L following stimulation with PMA plus ionomycin and intracellular staining of pCD40L for NK cells (*A*) and CD8^+^CD44^hi^ T cells (*B*) are shown. Data are representative of two independent experiments.
(TIF)
