# Supplementary figures and images for: Preformed CD40L Is Stored in Th1, Th2, Th17, and T Follicular Helper Cells as Well as CD4+8− Thymocytes and Invariant NKT Cells but Not in Treg Cells
Source: PLoS One. 2012 Feb 21;7(2):e31296. doi: 10.1371/journal.pone.0031296 (PMC3283616; doi:10.1371/journal.pone.0031296)

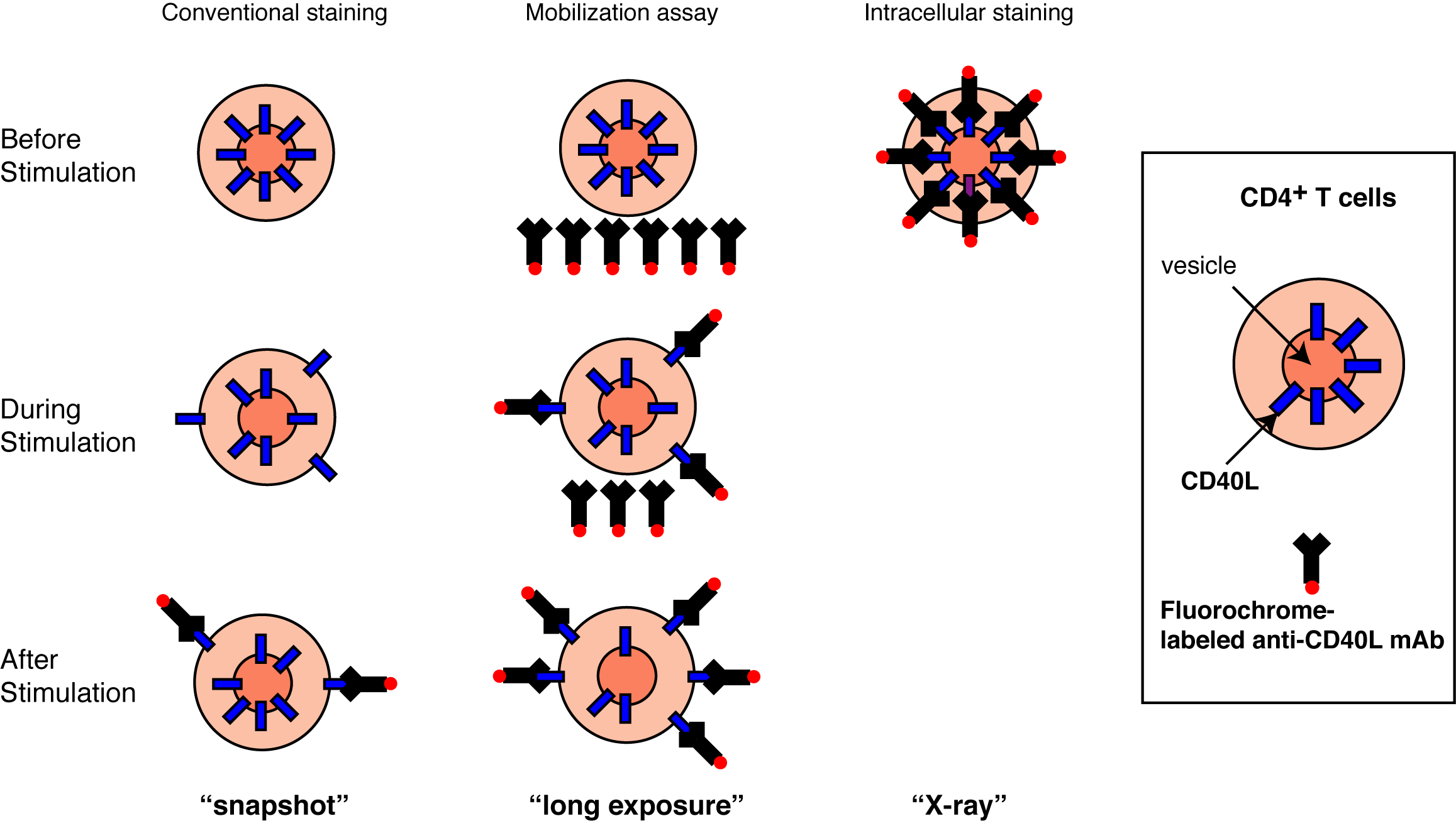

Supplement: Figure S1 — Schematic explanation of the mobilization assay. In the mobilization assay, fluorochrome-labeled anti-CD40L mAb is included in the culture during the activation of cells at 37°C. Compared to the “snap shot” nature of conventional staining at 4°C after completion of stimulation, the mobilization assay captures CD40L that has been delivered to the cell surface during stimulation while blocking CD40-dependent internalization, thereby providing the “long exposure” view of CD40L surface expression. By limiting the stimulation period to 30 minutes, we were able to exclude surface expression of de-novo CD40L made following stimulation (Koguchi, 2007). Intracellular staining can be seen as an “x-ray”, and is useful to distinguish defective mobilization of pCD40L from absence of stored pCD40L in cases where no mobilization of pCD40L is observed. (TIF) [file pone.0031296.s001.tif]

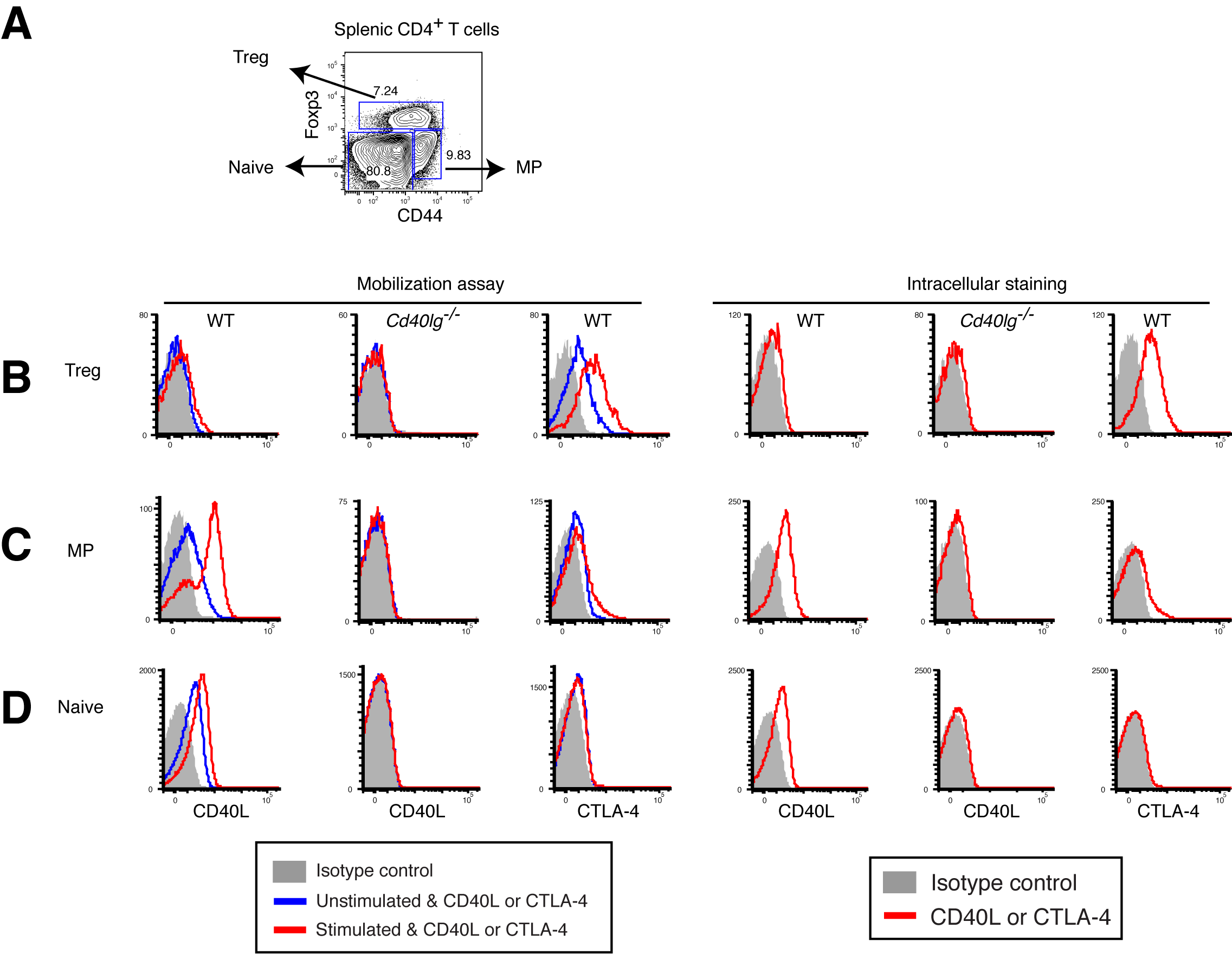

Supplement: Figure S2 — pCD40L expression in splenic CD4+ T cell subsets. A, Gating strategy for Treg cells, memory phenotype (MP) and naive CD4+ T cells. B–D, Mobilization of pCD40L upon stimulation with PMA plus ionomycin and intracellular staining of Treg cells (B), MP (C) and naive (D) CD4+ T cells. Data are representative of seven independent experiments. (TIF) [file pone.0031296.s002.tif]

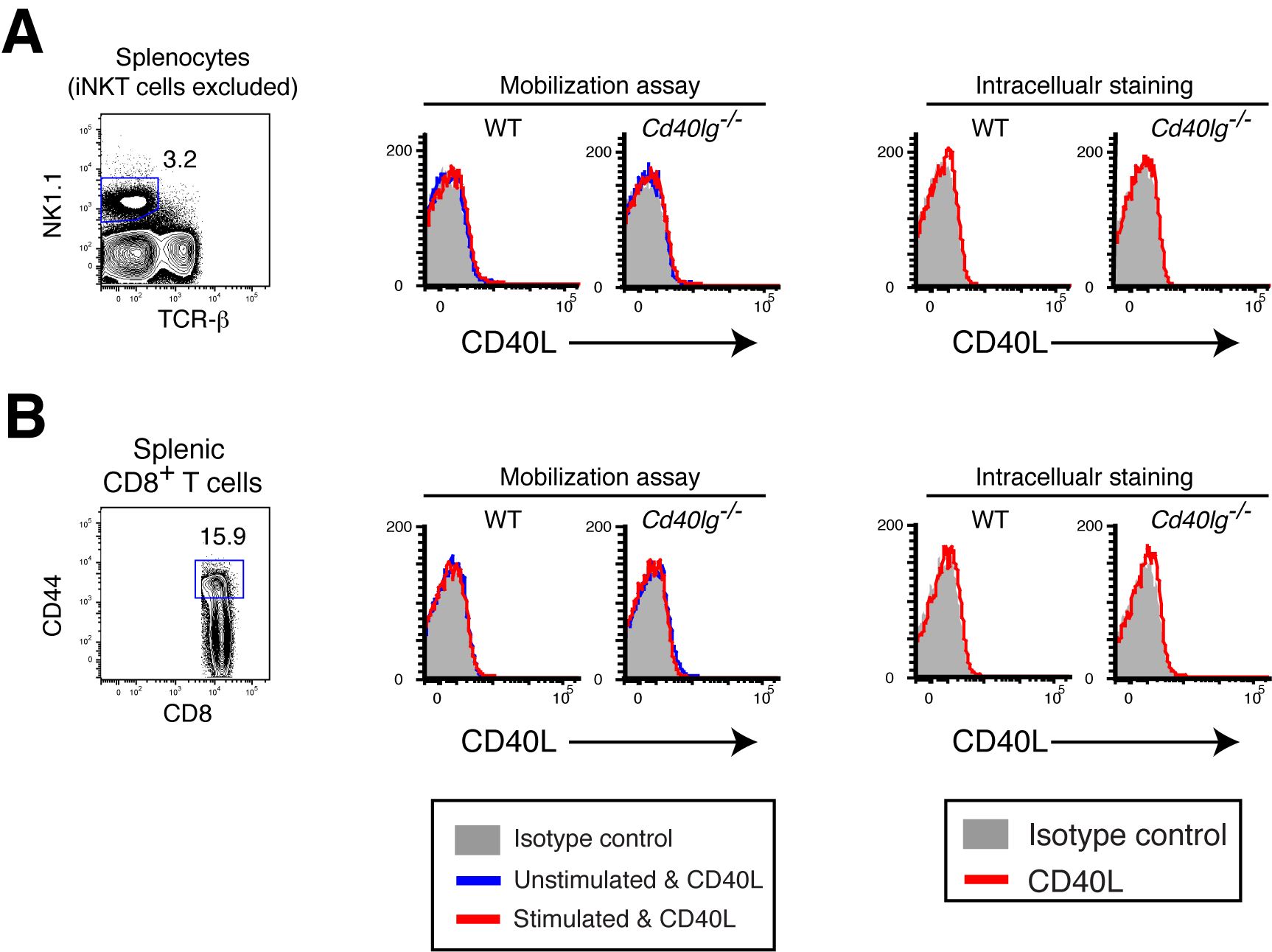

Supplement: Figure S3 — NK cells and CD8+ T cells do not have pCD40L. Gating strategies and the data for the mobilization of pCD40L following stimulation with PMA plus ionomycin and intracellular staining of pCD40L for NK cells (A) and CD8+CD44hi T cells (B) are shown. Data are representative of two independent experiments. (TIF) [file pone.0031296.s003.tif]
